# GP-DHT: A dual-head transformer with supervised contrastive learning for shared-gene pretraining and single-cell GRN prediction

**DOI:** 10.1371/journal.pone.0359164

**Published:** 2026-10-06

**Authors:** Shuai Yan, Qingzhi Yu, Wenfeng Dai, Xiang Cheng

**Affiliations:** School of Information Engineering, Jingdezhen Ceramic University, Jingdezhen, Jiangxi, China; Texas A&M University, UNITED STATES OF AMERICA

## Abstract

Gene Regulatory Networks (GRNs) are crucial for understanding cell fate decisions and disease mechanisms. However, inferring GRNs from single-cell RNA sequencing remains challenging due to noise, data sparsity, and species-specific distribution variations. We propose GP-DHT (Gene Pair–Dual Head Transformer), a single-cell GRN inference framework that pre-trains gene representations on a unified shared gene space using the BEELINE datasets of humans and mice. It models gene-cell expression relationships through a multi-relational heterograph and predicts transcription factor (TF)-target regulatory links using a dual-head Transformer. These two heads correspond to complementary task objectives: a classification head for regulatory link prediction and a projection head for gene pair-level supervised contrastive learning. This design enables the model to optimize both prediction accuracy and representation separability in a sparse single-cell setting. The human-mouse shared-gene stage is used to initialize expression-derived gene representations in a harmonized gene space. During dataset-wise supervised training, GenePairSupCon is applied within each training fold to regularize gene-pair representations, followed by fine-tuning and evaluation within each BEELINE dataset. To enhance reproducibility and interpretability, we further clarify the mapping of orthologous genes and gene symbol unification, TF-level data partitioning and leakage control, negative label sensitivity, and quantitative embedding quality assessment. Hard negative example experiments show that negative label construction has a substantial impact on task difficulty, while supervised contrastive learning significantly improves the separability of embeddings, as measured by Silhouette score, Davies-Bouldin index, Calinski-Harabasz score, and inter/intra distance ratio. GP-DHT can also recover prior-supported candidate regulatory modules and provide additional interpretable support through post-hoc pair-level attribution analysis on frozen predictors. In summary, these results support GP-DHT as a robust shared gene representation learning framework for dataset-level single-cell GRN prediction.

## 1 Introduction

Gene regulatory networks (GRNs) describe directed regulatory relationships between transcription factors (TFs) and their target genes and constitute a fundamental abstraction for understanding cell-fate decisions, developmental processes, and disease mechanisms. With the advent of single-cell technologies, GRN inference has progressively shifted from population-averaged expression analysis to cell-resolved modeling, enabling a more precise characterization of dynamic regulatory programs during cell-state transitions and lineage differentiation [1].

The rapid development of single-cell RNA sequencing (scRNA-seq) has provided unprecedented opportunities to investigate transcriptional heterogeneity at cellular resolution, thereby facilitating the identification of cell-type–specific and condition-dependent regulatory patterns [1,2]. Consequently, GRN inference based on single-cell data has become an important research direction in computational biology and systems biology, leading to a wealth of methodological summaries and reviews [3,4]. Recent advances have explored diverse modeling paradigms, including statistical frameworks [5,6], machine learning approaches [7,8], and deep neural architectures [7,9], to better capture nonlinear dependencies and context-dependent regulation.

Despite this progress, scRNA-seq–based GRN inference remains challenging. Compared to bulk RNA-seq, single-cell data are characterized by severe sparsity, technical noise, and zero inflation, which obscure true regulatory signals and exacerbate indirect associations [10]. Regulatory relationships often vary across cell types, developmental stages, and species, further complicating inference. Traditional GRN methods relying on population-averaged expression struggle to capture nonlinear regulatory effects and are influenced by indirect associations, with RNA-level interactions further complicating inferences [11]. These challenges are particularly pronounced in shared-gene pretraining settings, where expression distributions shift substantially and regulatory programs exhibit both conserved and species-specific components.

To address the limitations of traditional methods, deep learning approaches have been widely introduced to GRN inference in recent years to enhance representational capacity and modeling flexibility [12]. Neural network architectures can directly model the complex nonlinear dependencies between genes, forming an important branch of “deep learning for gene relationship inference” [13,14]. Representative models, such as DeepDRIM, employ deep architectures to capture cell-type–specific regulatory patterns, while recent benchmark studies have systematically evaluated inference reliability across diverse datasets [13]. Nevertheless, many existing deep learning methods primarily focus on pairwise gene associations [5,15] or local graph structures [16]and often lack mechanisms to explicitly incorporate global transcriptional context, limiting their interpretability and robustness.

Graph-based learning has emerged as a powerful paradigm for modeling structured biological data. Graph neural networks (GNNs) and attention mechanisms have been widely applied to single-cell analysis and GRN inference, enabling the integration of heterogeneous information and the modeling of complex dependencies [4]. Methods such as scGNN [17], GMFGRN [18] and HGATLink [19] leverage graph representations to improve regulatory edge prediction, while hypergraph and variational approaches further extend modeling capacity to higher-order regulatory modules [20]. In parallel, graph embedding and generative models for single-cell end-to-end representation learning are also being developed. For instance, a framework combining graph embedding and Gaussian mixture VAE has been used for end-to-end analysis of single-cell data, providing complementary insights into expression representation learning and structural modeling [21]. Furthermore, PMF-GRN uses variational inference to improve the robustness and stability of inference [22].

Beyond structural modeling, expression semantics enhancement and knowledge graph methods, like KEGNI, improve biological consistency and inference reliability, especially in low signal-to-noise scenarios [23]. Meanwhile, multi-omics integration has gradually become an important trend in GRN inference. For example, Graph-linked embedding has shown that integrating multi-omics can improve single-cell data integration and regulatory inference capabilities [24]. More general multi-omics integration frameworks have also been used for GRN prediction. In specific multimodal practices, SCENIC+ integrates ATAC-seq and TF motif data into the inference process to constrain candidate regulatory edges and reduce spurious regulation caused by co-expression [25]. Methods like SnapATAC provide foundational tools for the system analysis of single-cell ATAC-seq [26]. Similarly, large-scale functional genomics resources, such as ENCODE's cataloging of human/mouse regulatory elements, provide an important foundation for cross-species regulatory evidence integration and prior construction [27]. In addition, single-cell studies in specific biological systems have revealed the regulatory role of dynamic gene expression networks in B cell development and transformation, offering a reference for “network-level interpretability” [28].

While progress continues in single-species inference, the research focus has gradually expanded to the conservation and transferability of cross-species regulatory mechanisms. Early studies on cross-species network alignment have proposed statistical frameworks for cross-species network comparison [10]. Recently, cross-species single-cell representation learning methods have used shared latent spaces or foundational models to achieve expression alignment and knowledge transfer, such as GeneCompass, which constructs knowledge-driven cross-species foundational models to decipher universal regulatory mechanisms [29]. SATURN learns universal cell embeddings to reduce reliance on strict homologous mapping [30]. scPRINT, through pre-training on massive single-cell data, enhances the robustness and generalization of network predictions [31].

Despite significant progress, single-cell GRN inference with multi-dataset shared-gene representation learning still faces key challenges. First, simplifying continuous expression into traditional gene–gene graphs may lose expression-gradient information and weaken the capture of regulatory cues [2,24].In addition, negative-label construction substantially affects the difficulty and interpretation of GRN prediction. Work on negative sampling in graph learning provides useful methodological guidance for designing more reliable negative-label settings [32].

To address these challenges, we propose GP-DHT (GenePair-DualHeadTransformer), a single-cell GRN inference framework that combines heterogeneous graph learning, a dual-head Transformer module, and gene-pair-level supervised contrastive learning. Human and mouse datasets are used to construct a harmonized shared-gene pretraining space, while supervised GRN prediction is fine-tuned and evaluated within each BEELINE dataset. The dual-head design consists of a classification head for TF–target regulatory link prediction and a contrastive projection head for GenePairSupCon-based representation regularization. Accordingly, the role of multi-species data in this study is to improve shared-gene representation learning under sparse and noisy single-cell settings rather than to establish reliable direct species-to-species GRN transfer. We systematically evaluate GP-DHT on seven BEELINE benchmark datasets and focus the main claim on dataset-wise GRN prediction, robustness under negative-label uncertainty, and gene-pair representation separability.

Beyond reporting benchmark gains, we further investigate GP-DHT from three complementary perspectives. First, we examine how the classification head and the contrastive projection head contribute to regulatory prediction and gene-pair representation separability. Second, we analyze the influence of shared-gene pretraining, negative-label construction, and dataset-wise evaluation settings on model robustness. Third, we evaluate the stability and score-associated sensitivity of the auxiliary post-hoc attribution results to clarify the explanatory scope of the frozen predictor. Together, these analyses extend the study from predictive comparison to mechanism, robustness, and interpretation reliability.

## 2 Methods

### 2.1 Datasets

We evaluated seven single-cell RNA-seq datasets from the BEELINE benchmark suite, covering different human and mouse cell types, together with the corresponding cell-type-specific ChIP-seq-derived regulatory networks. Following the BEELINE preprocessing protocol, genes were filtered on raw count matrices before log transformation: a gene was retained only if it was detected (count > 0) in at least 1% of cells, with a minimum of 10 cells. We then selected the top 500 and 1,000 most variable genes according to expression-variance significance (Bonferroni-corrected p-value < 0.01) and variance ranking, resulting in 14 dataset configurations for evaluation. Zero entries were preserved in the expression matrices and handled during graph construction by masking and non-zero edge construction.

For label construction, TF–target pairs supported by the cell-type-specific ChIP-seq reference networks were treated as positive pairs. Following the TF-conditioned hard-negative sampling (HNS) rationale described in [33,34], negative candidates were constructed separately for each TF after excluding its reference-supported targets. Candidate negatives retained the same TF context as positive pairs but were paired with unlabeled candidate genes, thereby producing a more challenging negative set than uniform sampling from the full unlabeled TF–gene space.

GP-DHT directly uses the fixed, precomputed HNS label files supplied with the preprocessing data (dataset/ < cell type > /TFs+<gene-set size > / < dataset><gene-set size>shuffled_output.txt). For each dataset, all TF–gene pairs labeled as 0 in the corresponding HNS file are retained as the final negative set; GP-DHT performs no additional negative-pair subsampling, does not impose a fixed negative-to-positive ratio, and does not resample negatives during model training. Label 1 denotes a reference-supported regulatory edge, whereas label 0 denotes an HNS-derived putative negative. Consequently, the positive/negative counts reported in Table 1 reflect the complete records contained in the dataset-specific HNS files rather than a fixed 5–10 × negative-to-positive ratio imposed by GP-DHT. Because the reference networks are derived from ChIP-seq rather than perturbation experiments, label-0 pairs should be interpreted as putative negatives rather than experimentally verified non-regulatory interactions.

**Table 1:** Summary statistics of the scRNA-seq transcriptomes and corresponding ChIP-seq–derived regulatory networks.

Cell type	Number of cells	Number of TF genes	Genes (top 500/1,000)	Positive/negative sample ratio
hESC	759	34(34)	815(1260)	4545/26361(7084/40822)
hHEP	426	30(31)	874(1331)	9939/18471(15558/29299)
mDC	384	20(21)	443(684)	756/15644(1193/26527)
mESC	422	88(89)	977(1385)	29613/68859(42795/101296)
mHSC-E	1072	29(33)	691(1177)	11557/8830(21975/17724)
mHSC-GM	890	22(23)	618(1089)	7364/6518(14135/11878)
mHSC-L	848	16(16)	525(640)	4398/4545(5180/5876)

Shared-gene pretraining was performed over a harmonized human–mouse gene space before dataset-wise supervised fine-tuning. Human and mouse genes were matched using precomputed Ensembl BioMart-derived MAP_NAME resources after gene-symbol standardization, retaining only clean one-to-one shared orthologs and excluding ambiguous one-to-many or many-to-one mappings. In total, 875 clean shared orthologs were obtained from 2,457 human genes and 4,223 mouse genes. Detailed mapping statistics are provided in S1 Table. This shared-gene space was used only for representation initialization, while downstream GRN prediction and evaluation remained within each BEELINE dataset.

For dataset-wise evaluation, folds were generated at the TF level. All target-gene pairs associated with a held-out TF were assigned to the same test fold, preventing the same TF–target pair from appearing in both training and test partitions and avoiding supervised training on target pairs of the same held-out TF within a fold. The shared-gene pretraining stage used expression-derived gene embeddings and did not use regulatory labels from the held-out test fold.

Ethics statement. This study involved secondary analysis of publicly available, de-identified single-cell RNA-seq datasets from the BEELINE benchmark resources. No new human participants were recruited, and no new human specimens or tissue were collected by the authors. The authors did not have access to identifiable participant information. Therefore, ethics approval and informed consent were not required for this study.

### 2.2 Data preprocessing and feature extraction

To facilitate downstream representation learning and gene-pair prediction, GP-DHT first preprocesses the integrated human and mouse scRNA-seq data, constructs a gene-cell heterogeneous graph based on discretized expression relations, and then learns gene embeddings for downstream regulatory prediction. As illustrated in Fig 1, the overall framework consists of three major stages: data preprocessing and heterogeneous graph construction, relation-specific graph encoding and multi-relation fusion, and gene-pair modeling with dual-head prediction.

**Fig 1:**
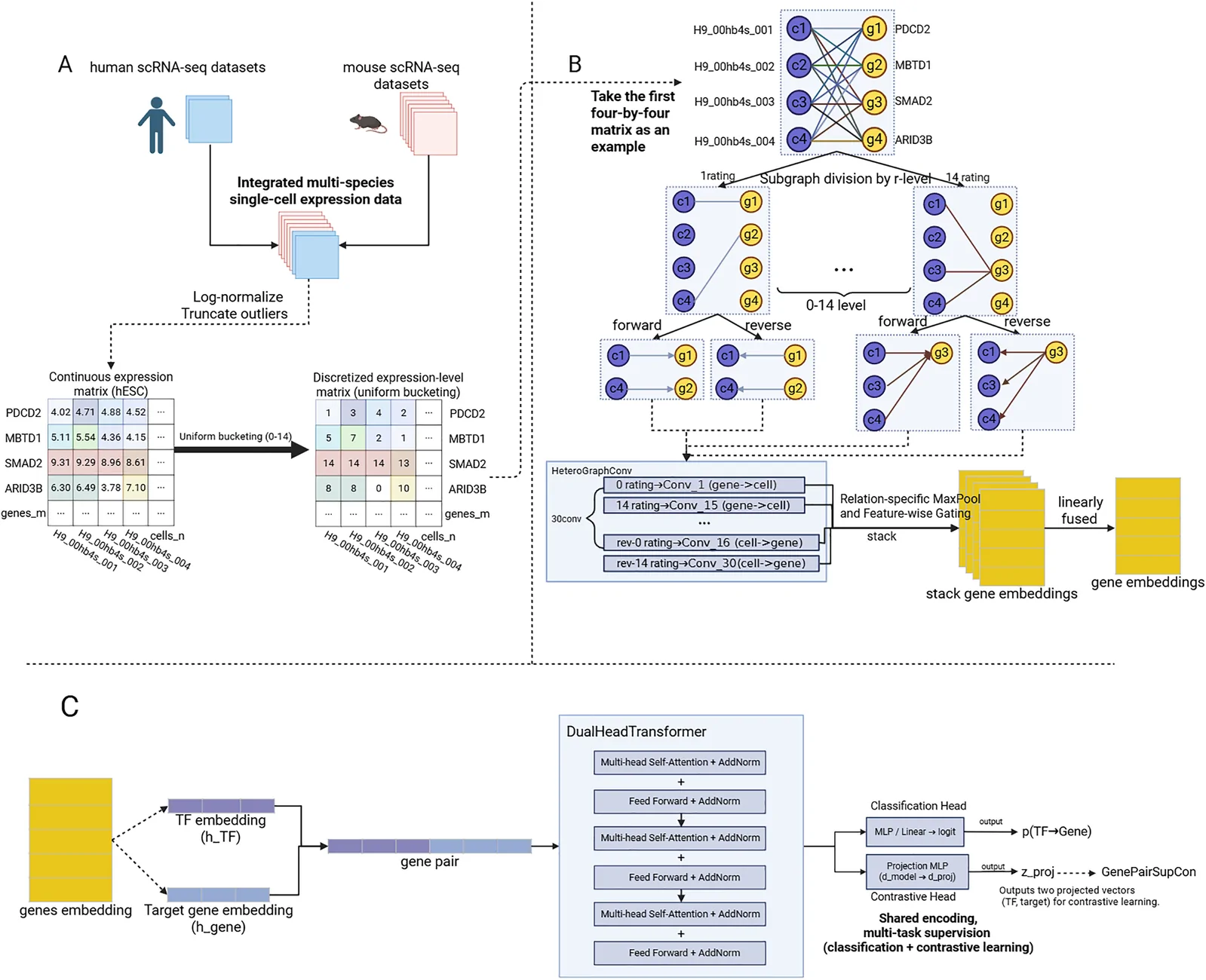
Overview of the GP-DHT framework. (A) Human and mouse scRNA-seq datasets are integrated, log-normalized, and truncated for outlier control. Continuous expression values are then discretized by uniform bucketing into ordinal expression-level relations, yielding a discretized gene-cell expression matrix for downstream heterogeneous graph construction. (B) A multi-relational gene-cell heterogeneous graph is constructed from the discretized expression matrix. For each relation type, transformed source-node messages are aggregated over the target node’s neighborhood by element-wise max pooling. The pooled message is subsequently modulated by a target-conditioned feature-wise gate. The relation-specific representations are then linearly fused to obtain the final gene embeddings. (C) For downstream GRN prediction, transcription factor and target-gene embeddings are paired and fed into a DualHeadTransformer. The learned pair representation is used by an MLP classification head for regulatory link prediction and by a projection head for GenePairSupCon-based supervised contrastive learning.

#### 2.2.1 Data processing.

Preprocessing is critical for ensuring the accuracy and stability of single-cell transcriptomic analyses. We perform several steps—gene filtering, log transformation, value clipping, and discretization—to enhance data quality. First, we filter genes on raw count matrices prior to log transformation: a gene is retained only if it is detected (count > 0) in at least 1% of cells in the dataset (with a minimum of 10 cells). This step removes extremely sparse genes that contribute little signal and may introduce noise. We then apply log transformation to the remaining gene expression values to compress the dynamic range and mitigate the impact of extreme values. Zero expressions are preserved to maintain sparsity. The log transformation is defined as:


$$\begin{array}{c} \begin{array}{c} \begin{array}{cc} {\text{x}}_{\text{ij}}^{\prime }= & \left\{ \begin{array}{cc} \text{ln}({\text{x}}_{\text{ij}}), & {\text{x}}_{\text{ij}}>\text{0}\\ \text{0}, & {\text{x}}_{\text{ij}}=\text{0} \end{array}\right.\;\;\text{i}\in \text{1},\text{2},\ldots ,\text{g};\;\text{j}\in \text{1},\text{2},\ldots ,\text{c} \end{array} \end{array} \end{array}$$ (1)

Next, extreme values are clipped. For each gene, we calculate the mean $\mu_{i}$ and standard deviation $\sigma_{i}$, defining the effective range $\left[\mu_{i}-\text{2}\sigma_{i},\mu_{i}+\text{2}\sigma_{i}\right]$. Values outside this range are clipped, reducing the influence of outliers. This range is computed as:


$$\begin{array}{c} \begin{array}{c} \begin{array}{c} \mu =\frac{\text{1}}{n}\sum_{i=\text{1}}^{n}\text{log}(x_{i}),\;\sigma =\sqrt{\frac{\text{1}}{n-\text{1}}\sum_{i=\text{1}}^{n}{\left(\text{log}(x_{i})-\mu \right)}^{\text{2}}} \end{array} \end{array} \end{array}$$ (2)

After clipping, we discretize expression values by mapping them to discrete levels based on predefined intervals. Out-of-range values are assigned to the lowest or highest level. The discretization process is formulated as:


$$\begin{array}{c} \begin{array}{c} {\text{x}}^{\prime }\prime_{\text{ij}}=\left\{ \begin{array}{cc} \text{k}, & \text{x}\prime_{\text{ij}}\in \text{Rang}{\text{e}}_{\text{ik}}\\ \text{1}, & \text{0}\leq \text{x}\prime_{\text{ij}}<\mu_{\text{i}}-\text{2}\sigma_{\text{i}}\\ \text{n}, & \text{x}\prime_{\text{ij}}>\mu_{\text{i}}+\text{2}\sigma_{\text{i}} \end{array}\right. \end{array} \end{array}$$ (3)

These preprocessing steps reduce noise, suppress extreme values, and transform the data into a suitable form for downstream modeling. The discretization process further helps extract meaningful features for regulatory network inference.

#### 2.2.2 Expression-level binning strategy.

We investigate two binning strategies for discretizing single-cell gene expression data: **Uniform Bucketing** and **Cumulative Bucketing**, each with different trade-offs in preserving continuity and producing discrete levels, affecting GRN inference.

**Uniform Bucketing:**

In this strategy, we first apply a log transformation to the gene expression values $x_{g}$, then uniformly partition the resulting range into K = 15 equal-width levels. The procedure is as follows:

Log transformation:

$$\begin{array}{c} {\text{l}}_{g}=\log(x_{g}) \end{array}$$ (4)

**Level Boundaries**: The mean μ, standard deviation σ, and min/max values of the log-transformed expression values are computed to define the level boundaries:


$$\begin{array}{c} \left[L,U]=[\text{max}(\mu -\text{2}\sigma ,t_{\text{min}}),\text{min}(\mu +\text{2}\sigma ,t_{\text{max}})\right] \end{array}$$ (5)

**Level Assignment**: Expression values are assigned to discrete levels based on their position in the defined range:


$$\begin{array}{c} \begin{array}{c} b_{uni}(x_{g})=\left\{ \begin{array}{cc} \text{0}, & if\;x_{g}=\text{0}\\ \text{min}\{K-\text{1},\text{max}(\text{0},\lfloor (\text{log}(x_{g})-L)/\text{w}\rfloor )\}+\text{1}, & if\;x_{g}>\text{0} \end{array}\right.\; \end{array} \end{array}$$ (6)

Here, K = 15 is the number of bins used throughout the paper.

**Continuous score normalization**:

$$\begin{array}{c} \begin{array}{c} r_{\text{uni}}\left(x_{g}\right)=\left\{ \begin{array}{cc} \frac{log\left(x_{g}\right)-L}{w} & \text{if }x_{g}>\text{0}\\ \text{9999} & \text{if }x_{g}=\text{0} \end{array}\right. \end{array} \end{array}$$ (7)

**Cumulative Bucketing:**

This strategy uses a pyramid-style binning, where each gene expression value is assigned to multiple levels, including all levels up to its own. The key steps are:

**Level Calculation**: We first compute the level for each gene using uniform bucketing. Then, the effective number of levels is determined by:


$$\begin{array}{c} \begin{array}{c} L^{⋆}=b_{\text{uni}}(x) \end{array} \end{array}$$ (8)

**Level Expansion**: Each gene–cell pair is assigned to all levels up to $L^{⋆}$, creating additional edges in the graph:


$$\begin{array}{c} \begin{array}{c} E=\overset{L^{⋆}}{\underset{l=\text{1}}{⋃}}\left(g\to c),(c\to g\right) \end{array} \end{array}$$ (9)

**Continuous score normalization**: Similar to uniform bucketing, values are computed for each gene across all levels:


$$\begin{array}{c} \begin{array}{c} r_{\text{cumulative}}\left(x_{g}\right)=\left\{ \begin{array}{cc} \frac{\text{log}\left(x_{g}\right)-L}{w} & \text{if }x_{g}>\text{0}\\ \text{9999} & \text{if }x_{g}=\text{0} \end{array}\right. \end{array} \end{array}$$ (10)

**Computational Complexity**

**Uniform Bucketing**: Each non-zero entry leads to one edge, resulting in 2M edges, where M is the number of non-zero elements.

**Cumulative Bucketing**: Each non-zero entry connects to multiple level relations, resulting in O(KM) edges, where K is the number of bins.

Both strategies increase graph complexity and memory consumption with larger K, but provide richer relational information to improve prediction accuracy.

In graph construction, we mask zero-expression entries and create gene–cell edges only from non-zero expression pairs; zero entries therefore do not introduce edges and are excluded from downstream message passing.

#### 2.2.3 Gene–cell representation learning: Embedding model construction.

We propose a heterogeneous-graph representation learning framework to model regulatory relationships between genes and cells, treating genes and cells as node types, with gene–cell pairs forming edges. Edge types are defined by discretized expression levels, with reverse edges added to enhance information propagation.

This design captures multi-dimensional expression relationships. Genes and cells are encoded using one-hot vectors. During training, node features are propagated and aggregated using relation-specific message passing, element-wise max pooling, and feature-wise gating mechanisms to learn embeddings.

To capture expression-level-specific interactions, we use an independent message-passing pathway for each relation type. The implemented operation is a relation-specific max-pooling layer followed by target-conditioned feature-wise message gating. It is not standard GAT-style neighbor attention because it does not assign and normalize one scalar attention coefficient across individual neighboring nodes.

For each edge type r, representing a discretized expression level and direction, a directed pair (u, v) contains source node u and target node v. Let h_u and h_v denote their input features, and let $c_{u}$ and $c_{v}$ denote the endpoint context vectors stored on the graph. The source feature is projected and a target-derived gating query is constructed as follows:


$$\begin{array}{c} \begin{array}{c} z_{u}^{\left(r\right)}=W_{r}h_{u},{\;q}_{v}^{\left(r\right)}=\text{ reshape}\left[(W_{k}h_{v})⊙c_{v}\right] \end{array} \end{array}$$ (11)

where $W_{r}$ and $W_{k}$ are learnable transformation matrices, $c_{v}$ is the target-side context vector, and reshape divides the projected representation across the configured feature groups.

For target node v, each projected source feature is modulated by its source-side context vector $c_{u}$, passed through ReLU, and aggregated over the relation-specific neighborhood $N_{r}(v)$ by element-wise max pooling:


$$\begin{array}{c} \begin{array}{c} m_{v}^{\left(r\right)}=\underset{u\in N_{r}(v)}{\text{MaxPool}}\;\text{ReLU}\left[z_{u}^{\left(r\right)}⊙\text{Dropout}(c_{u})\right] \end{array} \end{array}$$ (12)

A feature-wise gate is then computed solely from the target-derived query $q_{v}^{\left(r\right)}$. After L2 normalization, element-wise self-product, absolute value, and temperature scaling by τ, Softmax is applied across the feature dimension:


$$\begin{array}{c} \begin{array}{c} \alpha_{v}^{\left(r\right)}={\text{Softmax}}_{\text{feature}}\left(\frac{\left|\text{Norm}\left(q_{v}^{\left(r\right)}\right)⊙\text{Norm}\left(q_{v}^{\left(r\right)}\right)\right|}{\tau }\right) \end{array} \end{array}$$ (13)

The resulting vector represents target-conditioned feature-wise gates on the max-pooled source message. The relation-specific updated representation is computed by element-wise modulation. Thus, the weights are normalized across feature dimensions rather than neighboring nodes:


$$\begin{array}{c} \begin{array}{c} h_{v}^{\prime (r)}=m_{v}^{\left(r\right)}⊙\alpha_{v}^{\left(r\right)} \end{array} \end{array}$$ (14)

The relation-specific representations are then concatenated across all relation types and projected back to the target dimension $d_{out}$ through a linear layer, yielding the final representation of node v:


$$\begin{array}{c} \begin{array}{c} {\text{h}}_{\text{v}}={\text{W}}_{\text{o}}({∥}_{(}\text{r}\in \text{R}){\text{h}}_{\text{v}}^{(\text{r}{)}^{\prime }})+b \end{array} \end{array}$$ (15)

The heterogeneous graph encoding is performed in parallel across all gene and cell nodes. After one layer of relation-specific message passing and multi-relation fusion, the model generates gene and cell embedding matrices, respectively. These embeddings capture multi-level gene–cell expression relations and provide inputs for the downstream dual-head Transformer decoder used for regulatory link classification and supervised contrastive regularization. Learned during the pooled shared-gene representation stage, these embeddings are further used to initialize dataset-wise fine-tuning for GRN prediction.

### 2.3 Gene regulatory prediction via a dual-head transformer

After obtaining embedding representations for genes and cells, a key step in single-cell GRN inference is how to effectively map these representations to reliable gene regulatory relationships. To this end, we design a dual-head Transformer–based regulatory prediction module with two task-specific heads: a classification head for regulatory link prediction and a projection head for supervised contrastive learning. The two heads correspond to prediction and representation-regularization objectives rather than two separate local/global architectural branches. This design is motivated by the biological nature of transcriptional regulation: true regulatory relationships depend not only on direct interactions between transcription factors and target genes, but are also jointly modulated by the broader transcriptional environment, including cell type, developmental stage, and species background.

#### 2.3.1 Design rationale: Dual-head Transformer with classification and contrastive heads.

In GRN inference, accurately modeling regulatory relationships between gene pairs is crucial. To jointly optimize regulatory prediction and gene-pair representation learning, we propose a dual-head Transformer architecture. The dual-head design in GP-DHT refers to two task-specific heads built on top of the Transformer-encoded gene-pair representation: a classification head for regulatory link prediction and a projection head for supervised contrastive learning. The classification head outputs the final TF–target regulatory probability, whereas the projection head is used during training to compute the GenePairSupCon loss. This design improves performance and robustness by jointly optimizing regulatory prediction and gene-pair representation separability, especially under sparse and human-mouse shared-gene pretraining settings.

The regulatory prediction head, also referred to as the classification head, focuses on predicting whether a regulatory relationship exists between a given TF–target pair. It takes the Transformer-encoded gene-pair representation as input and outputs the final regulatory probability.


$$\begin{array}{c} \begin{array}{c} {\text{H}}_{\text{class}}=\text{ClassHead}({\text{X}}_{\text{genepair}}) \end{array} \end{array}$$ (16)

The contrastive learning head, implemented as a projection head, is used only during training. It maps Transformer-encoded gene-pair representations into a contrastive embedding space, where GenePairSupCon encourages gene pairs with the same label to be closer and pairs with different labels to be farther apart. This objective helps the model learn more separable and robust gene-pair representations under sparse and human-mouse shared-gene pretraining settings.


$$\begin{array}{c} \begin{array}{c} {\text{H}}_{\text{contrast}}=\text{ContrastHead}({\text{X}}_{\text{genepair}}) \end{array} \end{array}$$ (17)

#### 2.3.2 Transformer encoder architecture.

We employ a Transformer encoder with three layers, each consisting of multi-head self-attention, a position-wise feed-forward network (FFN), and layer normalization. This architecture enables the model to capture complex nonlinear dependencies between gene pairs, discovering latent associations without relying on a predefined graph topology.

The gene-pair embedding matrix $X_{\text{genepair}}$ is fed into the encoder. In each layer, self-attention models interactions among input features, enhancing the expressiveness of the learned representations. Multi-head self-attention computes the weighted sum of the value matrix V using query Q and key K matrices, as shown below:


$$\begin{array}{c} \begin{array}{c} \text{Attention}\left(Q,K,V\right)=\text{softmax}\left(\frac{Q^{⊤}K}{\sqrt{d_{k}}}\right)V \end{array} \end{array}$$ (18)

Next, the feed-forward network (FFN) applies a GELU activation function:


$$\begin{array}{c} \begin{array}{c} \text{FFN}\left(x\right)=\text{GELU}\left(xW_{1}+b_{1}\right)W_{2}+b_{2} \end{array} \end{array}$$ (19)

The output of each layer is normalized using layer normalization:


$$\begin{array}{c} \begin{array}{c} \text{LayerNorm}\left(x\right)=\gamma \cdot \frac{x-\text{E}\left[x\right]}{\text{Var}\left[x\right]}+\epsilon +\beta \end{array} \end{array}$$ (20)

Finally, the encoder output h is mapped to a 256-dimensional representation using a fully connected layer with GELU activation and layer normalization:


$$\begin{array}{c} \begin{array}{c} f=\text{LayerNorm}\left(\text{GELU}\left(hW+b\right)\right) \end{array} \end{array}$$ (21)

This design effectively captures complex dependencies between gene pairs.

#### 2.3.3 Robust representation constraints via contrastive learning.

GRN inference from single-cell transcriptomic data faces challenges such as data sparsity, noise, incomplete labels, and dataset-specific expression shifts. Conventional supervised learning methods struggle in these settings, often leading to overfitting and limited generalization. To address this, we introduce contrastive learning to improve model robustness and representation separability.

Contrastive learning optimizes gene-pair embeddings by maximizing the similarity of positive pairs (gene pairs with regulatory relationships) and minimizing that of negative pairs (non-regulatory gene pairs). This approach helps the model better capture regulatory signals between genes under sparse and noisy single-cell settings.

Within the dual-head Transformer, the projection head maps Transformer-encoded gene-pair representations into a contrastive embedding space, which optimizes gene-pair embeddings by computing pairwise similarities. The contrastive loss, based on cosine similarity, encourages higher similarity for positive pairs and lower similarity for negative pairs. The loss is defined as:


$$\begin{array}{c} \begin{array}{c} L_{\text{SupCon}}\left(h_{i},h_{j}\right)=-log\frac{exp\left(\text{sim}\left(h_{i},h_{j}\right)/\tau \right)}{\sum_{k\neq i}exp(\text{sim}(h_{i},h_{k})/\tau )} \end{array} \end{array}$$ (22)

where $\text{sim}(h_{i},h_{j})$ is the cosine similarity between gene-pair embeddings, and τ is a temperature parameter controlling the sharpness of the similarity distribution.

#### 2.3.4 Joint optimization of classification and contrastive losses.

To improve robustness and generalization under sparse conditions, we introduce a weighted loss function that combines classification and contrastive losses. This helps the model predict regulatory relationships while refining gene-pair representations using contrastive learning.

We also apply data augmentation by injecting Gaussian noise into the training data, increasing diversity and helping the model learn stable features. Specifically, Gaussian noise is added to the input features 𝐗, generating augmented data with controlled noise strength.

The model is trained using binary cross-entropy loss for regulatory predictions and contrastive loss for robustness. During training, we apply the same masking strategy as in graph construction, so that only non-zero gene–cell entries contribute to the learning signal, while zero entries are ignored. The total loss is:


$$\begin{array}{c} \begin{array}{c} L_{\text{total}}=L_{\text{BCE}}+\lambda \cdot L_{\text{SupCon}} \end{array} \end{array}$$ (23)

where $L_{\text{BCE}}$ is the binary cross-entropy loss, $L_{\text{SupCon}}$ is the supervised contrastive loss, and λ is a weighting factor.

We compute losses on both the original and augmented data and average them to fully utilize augmented samples during training.

The final training objective combines both losses:


$$\begin{array}{c} \begin{array}{c} L_{\text{total}}=\text{0}.\text{6}\cdot L_{\text{BCE},\text{ total}}+\text{0}.\text{4}\cdot L_{\text{SupCon},\text{ total}} \end{array} \end{array}$$ (24)

Hyperparameters, including learning rate and weight decay, were tuned on a validation set, with an embedding dimension of 256 yielding optimal performance.

To better understand the contribution of each component beyond aggregate performance, we further conduct targeted ablation and stress-test analyses under varying sparsity, noise, and training-data regimes in the experimental section.

#### 2.3.5 Post-hoc pair-level attribution analysis.

To provide an additional interpretability layer without modifying the main predictive pipeline, we further performed an auxiliary post-hoc pair-level attribution analysis on the frozen GP-DHT predictor. It is important to distinguish the graph levels involved in the predictive and explanatory procedures. The expression-level relation subgraphs described in Section 2.2 belong to the upstream gene-cell heterogeneous graph and are used for multi-relational message passing and gene embedding learning. By contrast, the attribution analysis operates on downstream pair-centered local context graphs, in which each candidate TF-target pair is treated as the focal pair and embedded within a compact local neighborhood for attribution analysis. Node features in these local context graphs are initialized using the gene embeddings learned from the upstream heterogeneous graph encoder. Accordingly, this analysis does not reconstruct a new global regulatory topology; instead, it assigns edge-level importance weights within the original local context graph to support interpretation of the model prediction. The overall workflow of this auxiliary post-hoc attribution procedure is illustrated in Fig 2.

**Fig 2:**
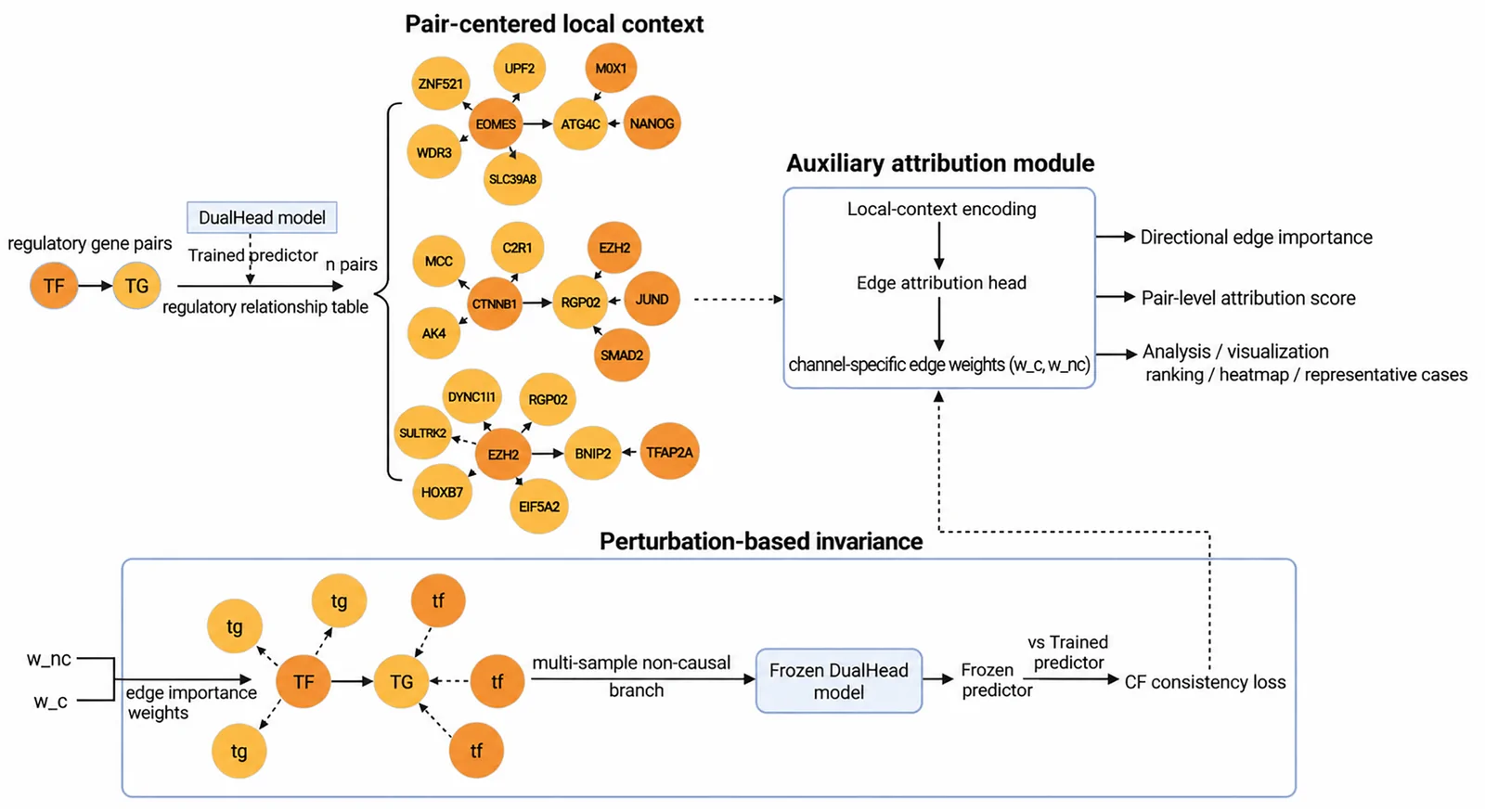
Workflow of the auxiliary post-hoc attribution analysis on the frozen GP-DHT predictor. For each candidate TF-target pair, a pair-centered local context graph is constructed around the focal pair and passed to an auxiliary attribution module for local-context encoding and edge attribution. The module produces channel-specific edge weights, from which directional edge importance and pair-level attribution scores are derived. To improve the stability of the attribution results, a perturbation-based invariance procedure further regularizes the auxiliary module by enforcing consistency between the frozen predictor and perturbed variants. The resulting attribution scores are used only for post-hoc analysis and visualization, including ranking, heatmaps, and representative case studies.

The attribution procedure is conducted after the main GP-DHT model has been trained and the best checkpoint has been selected according to validation performance. The predictor is then frozen, and an auxiliary attribution module is optimized on top of the pair-centered local context graphs. For each candidate pair, the module performs local-context encoding and edge attribution to produce channel-specific edge weights, from which directional edge importance is obtained. These edge-level quantities are further aggregated into a pair-level attribution score that reflects the overall explanatory support for the focal TF-target prediction. Because the task model remains fixed throughout this stage, the objective of the auxiliary module is to interpret the decision basis of the trained predictor rather than to re-optimize the predictor itself.

To improve the stability of the attribution results, the auxiliary module is trained with a perturbation-based invariance objective implemented through consistency regularization on perturbed variants under the frozen predictor. Under this formulation, edges assigned larger weights can be interpreted as directional relations that more consistently support the prediction across perturbed variants of the local context. The resulting directional edge importance and pair-level attribution scores are used only for post-hoc analysis, including ranking, visualization, and representative case studies. These outputs should therefore be interpreted as prediction-level attribution for the frozen predictor, rather than as direct evidence of biological interventional causality.

In addition to qualitative visualization, we further assess the attribution outputs using stability diagnostics and score-associated sensitivity analyses to examine how consistently the post-hoc explanation reflects the decision behavior of the frozen predictor.

## 3 Experiments and results

To evaluate GP-DHT in its intended setting, we use pooled shared-gene pretraining over the harmonized human–mouse gene space and report dataset-wise fine-tuning and evaluation on the BEELINE benchmarks. AUROC is used to summarize overall discrimination performance, whereas AUPRC provides an evaluation that is more informative under class imbalance. To further assess the recovery of high-confidence regulatory edges at the top of the ranked prediction list, we additionally report Precision@K. For each test fold, K is defined as the number of positive reference edges in that fold, and Precision@K is calculated as the proportion of positive reference edges among the K highest-scoring TF–target pairs. Fold-level Precision@K values are summarized as mean ± SD. Because direct species-to-species transfer is not the established claim of this study, the Results section focuses on within-dataset prediction, negative-label sensitivity, embedding-quality analysis, and model robustness.

We use a three-fold cross-validation protocol based on TF-level splitting. In each fold, approximately one third of TFs and all corresponding TF-target pairs are held out for testing, while the remaining TFs are used for training and validation. Instead of averaging across folds, we aggregate all test samples for a more stable evaluation, reducing the impact of small-sample fluctuations.

For model optimization, we use the Adam optimizer with a batch size of 512. Training stops after 200 iterations or if parameters are not updated for 10 consecutive steps. We compare GP-DHT with several baseline methods: HGATLink [17,19] (combining heterogeneous graph attention networks with a Transformer to improve GRN inference), GMFGRN [18] (leveraging heterogeneous information and heterogeneous-graph embeddings to reduce false positives), IGEGRN [20] (learning embeddings via GraphSAGE and producing final predictions after three convolutional layers), GNN-Link [35] (a graph neural network-based link prediction method for inferring gene regulatory relationships from single-cell expression data), and GATCL [36] (performing GRN inference by learning graph attention using convolutional layers instead of weight matrices).The overall performance comparison across the seven scRNA-seq datasets is summarized in Fig 3.

**Fig 3:**
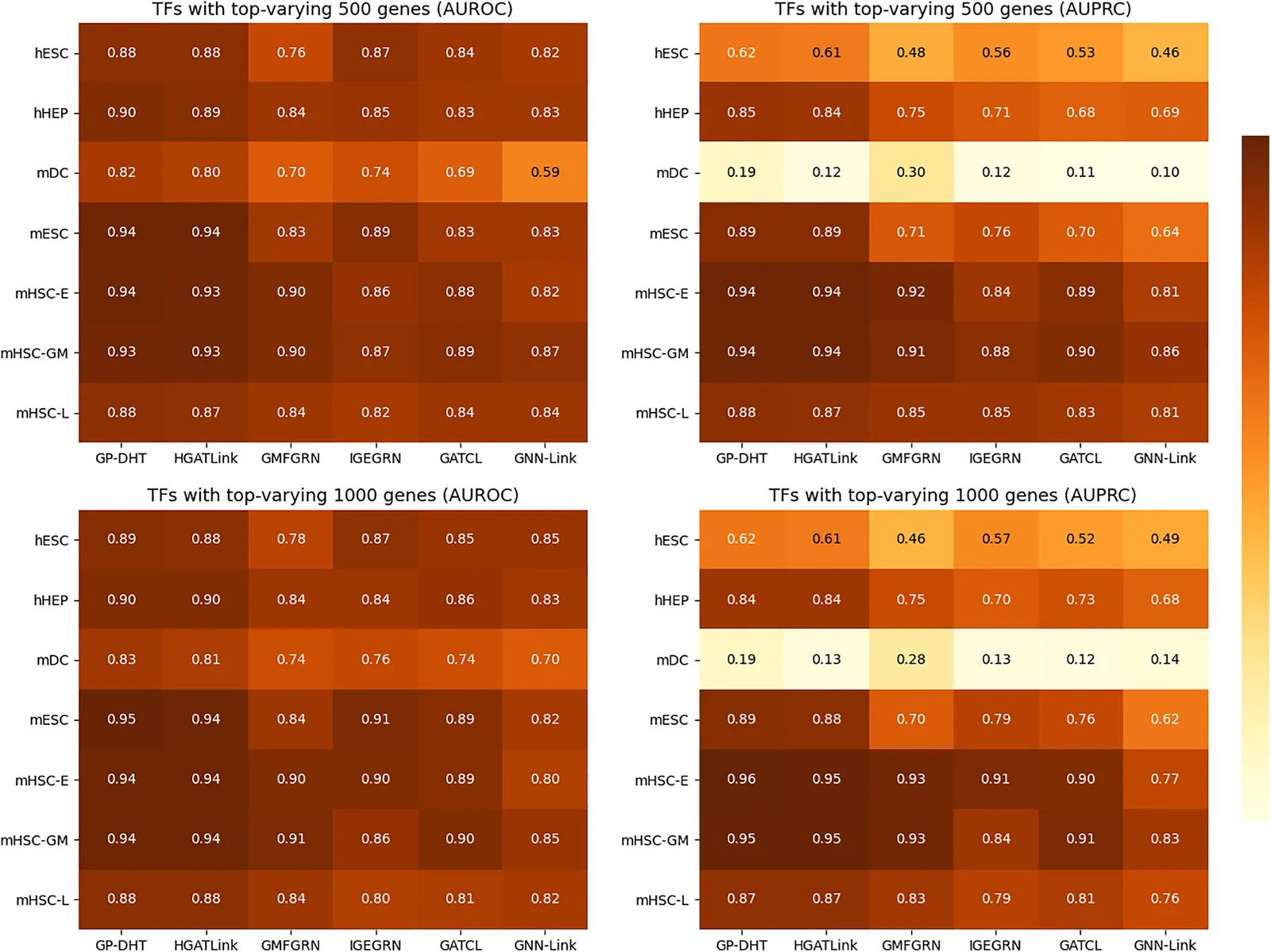
Performance comparison of GP-DHT with five baseline methods across seven scRNA-seq datasets. AUROC (left) and AUPRC (right) are reported for models trained on the top 500 most variable genes (top row) and top 1,000 most variable genes (bottom row). Baselines include HGATLink, GMFGRN, IGEGRN, GATCL, and GNN-Link, implemented following the original descriptions (see Methods). Scores are reported on the same train/validation/test splits for all methods (see Methods). Each subplot corresponds to one dataset (dataset names shown in panel labels).

In addition to predictive performance, we further conducted an auxiliary post-hoc attribution analysis on the frozen GP-DHT model to summarize edge-level importance patterns for representative TF–target predictions.

### 3.1 Negative-label sensitivity analysis

Although the main pipeline uses TF-conditioned HNS labels, the resulting negative-label pairs remain putative because the ChIP-seq-derived reference networks are incomplete and do not provide experimentally verified biological negatives. We therefore conducted auxiliary sensitivity analyses under alternative negative-label settings as stress tests for label uncertainty. Across the 0%, 5%, and 10% auxiliary settings, random putative negatives yielded AUROC values of 0.8827–0.8865 and AUPRC values of 0.8053–0.8091, whereas the stricter hard-negative settings yielded AUROC values of 0.8312–0.8325 and AUPRC values of 0.7538–0.7551. The consistent decreases in both AUROC and AUPRC show that hard-negative construction creates a more challenging evaluation setting under class imbalance and that performance obtained using easier random negatives should be interpreted cautiously. Precision@K showed a consistent pattern: random putative negatives achieved values of 0.735–0.749, whereas hard negatives yielded values of 0.670–0.687, corresponding to an average decrease of approximately 0.063. This result further indicates that hard-negative construction affects not only global discrimination and class-imbalance-sensitive performance, but also the recovery of high-confidence regulatory edges at the top of the ranked prediction list. Full mean ± SD results are provided in S2 Table.

### 3.2 Quantitative embedding-quality analysis for GenePairSupCon

To provide quantitative support beyond PCA visualization, we compared contrastive embeddings learned with and without GenePairSupCon using multiple embedding-quality metrics. As summarized in Table 2, GenePairSupCon substantially improved representation geometry: the Silhouette score increased from 0.1893 ± 0.0319 to 0.3619 ± 0.0432, the Davies-Bouldin index decreased from 1.8443 ± 0.1521 to 1.1633 ± 0.0975, the Calinski-Harabasz score increased from 136.0 ± 24.1 to 282.8 ± 26.4, and the inter/intra distance ratio increased from 1.3081 ± 0.1520 to 2.4893 ± 0.2504. These results show that the supervised contrastive objective primarily improves the separability and compactness of gene-pair representations, while predictive performance remains jointly determined by the classification and contrastive objectives.

**Table 2:** Quantitative embedding-quality comparison with and without GenePairSupCon based on contrastive embeddings.

Condition	Silhouette	Davies-Bouldin	Calinski-Harabasz	Inter/Intra ratio	AUROC	AUPRC
With GenePairSupCon	0.3619 ± 0.0432	1.1633 ± 0.0975	282.8 ± 26.4	2.4893 ± 0.2504	0.8848 ± 0.0113	0.8156 ± 0.0119
Without GenePairSupCon	0.1893 ± 0.0319	1.8443 ± 0.1521	136.0 ± 24.1	1.3081 ± 0.1520	0.8807 ± 0.0138	0.8125 ± 0.0135

### 3.3 Complementary heads

To attribute GP-DHT’s gains, we ablate the Dual-head decoder and GenePairSupCon separately. Removing the contrastive head/objective consistently degrades performance, indicating that GenePairSupCon is not merely auxiliary: it improves representation separability by pulling true regulatory pairs together and pushing non-regulatory pairs apart, which stabilizes the ranking of weak edges and long-tail TF/TG pairs under sparsity, and mitigates degradations caused by dropout noise and dataset-level shifts.

Complementarily, the dual-head Transformer module combines a classification head and a contrastive projection head on the Transformer-encoded gene-pair representation. The classification head learns the decision boundary for TF–target link prediction, whereas the projection head introduces GenePairSupCon to improve intra-class compactness and inter-class separation in the gene-pair embedding space. Since the two heads optimize complementary objectives—regulatory prediction and representation regularization—they are not interchangeable; their combination forms a key advantage of GP-DHT in sparse and heterogeneous dataset settings.

### 3.4 Effect of the binning strategy

We evaluated two binning strategies, Uniform Bucketing and Cumulative Bucketing. All experiments were conducted under the same hyperparameter configuration, and model performance was assessed using AUC and AUPR. The results show that Uniform Bucketing achieves higher performance than Cumulative Bucketing; therefore, we adopt Uniform Bucketing as the default binning scheme in the final model.

As shown in Table 3, the Uniform Bucketing strategy achieves higher AUC and AUPRC on most datasets. This is mainly because cumulative bucketing introduces a large number of overlapping edges, which amplifies redundant signals from highly expressed genes and causes local noise to be aggregated multiple times. In contrast, uniform bucketing preserves only mutually exclusive levels, reducing the number of edges while retaining discretized information about expression gradients, thereby improving the representation efficiency of the heterogeneous graph.

**Table 3:** Comparison of different binning strategies for gene expression discretization.

Dataset	Uniform Bucketing		Cumulative Bucketing	
	AUC	AP	AUC	AP
hESC1000	0.89	0.62	0.86	0.54
hHEP1000	0.90	0.84	0.85	0.72
mDC1000	0.83	0.19	0.78	0.12
mESC1000	0.95	0.89	0.93	0.86
mHSC-E1000	0.94	0.96	0.92	0.93
mHSC-GM1000	0.94	0.95	0.89	0.90
mHSC-L1000	0.88	0.87	0.86	0.85
hESC500	0.88	0.62	0.86	0.55
hHEP500	0.90	0.85	0.85	0.74
mDC500	0.82	0.19	0.79	0.12
mESC500	0.94	0.89	0.93	0.87
mHSC-E500	0.94	0.94	0.91	0.93
mHSC-GM500	0.93	0.94	0.92	0.92
mHSC-L500	0.88	0.88	0.86	0.85

The results in Table 3 indicate that uniform bucketing yields better AUC and AUPRC on the majority of datasets. By comparison, although cumulative bucketing introduces richer relational information at the expression level, it also substantially increases edge redundancy and magnifies the noise accumulation effect associated with highly expressed genes.

By maintaining the exclusivity of expression levels, uniform bucketing effectively preserves expression-gradient information while controlling graph-structure complexity, achieving a more favorable trade-off between representational efficiency and predictive performance.

### 3.5 Visualization analysis

To validate the predictive performance and biological interpretability of GP-DHT, we generate three types of visualizations for each dataset: Precision–Recall (PR) curves, Top-K regulatory network graphs, and TF–target gene (TF–TG) heatmaps. These visualizations offer insights into model performance, network structure, and biological relevance.

First, the PR curves (Fig 4) illustrate how precision and recall vary under different decision thresholds. Across datasets, the average precision (AUPRC) consistently exceeds the random-classification baseline, with relatively narrow 95% confidence intervals, indicating that GP-DHT maintains stable discriminative capability across multiple cell types and species. Notably, on stem-cell datasets such as hESC1000 and mESC1000, the AUPRC exceeds 0.55, demonstrating the model’s effectiveness in identifying true regulatory relationships.

**Fig 4:**
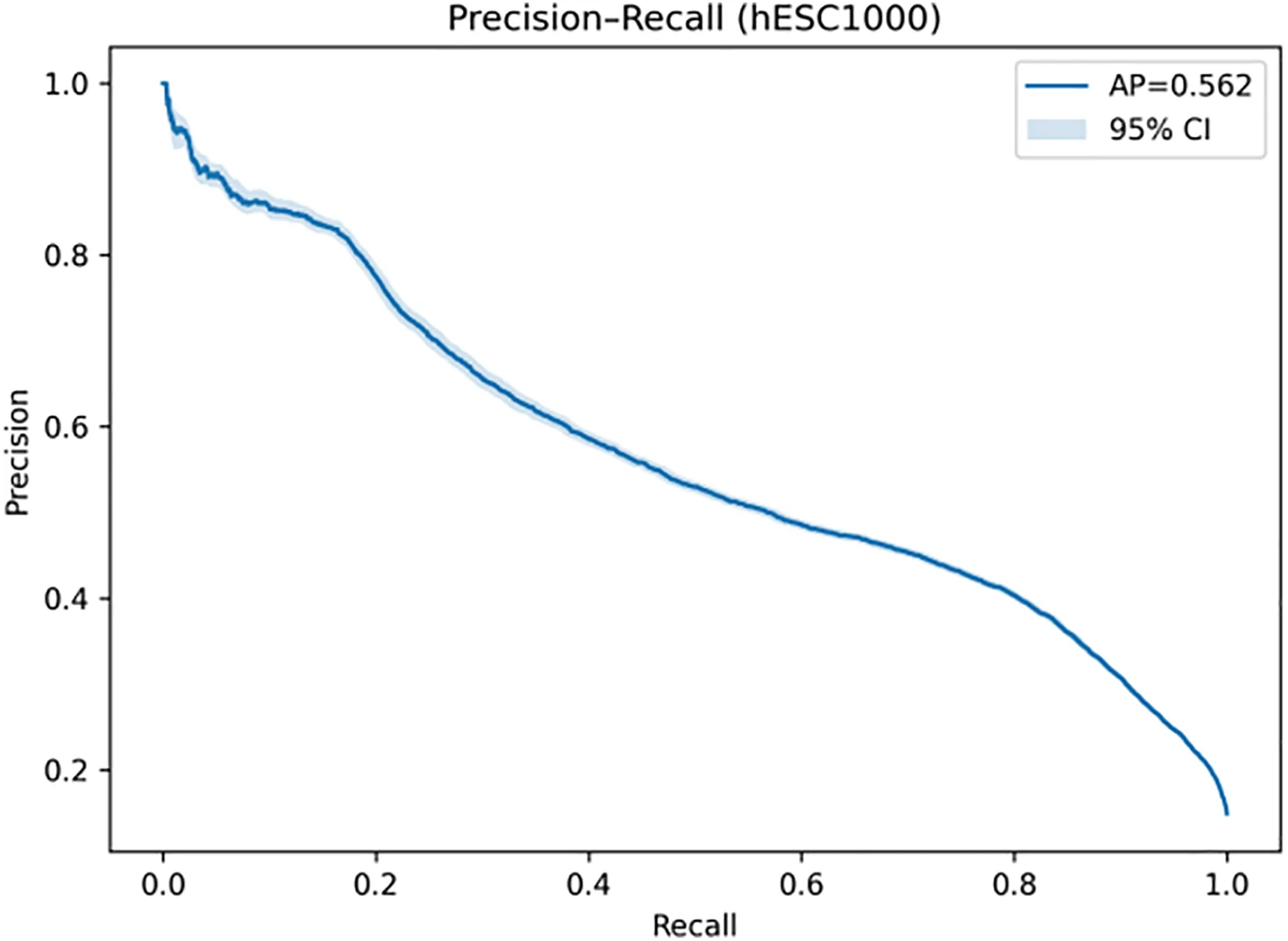
Precision–recall (PR) curves illustrating the performance of GP-DHT across different single-cell datasets.

Second, the Top-K regulatory network graphs (Fig 5) visualize the top 100 transcriptional regulatory edges with the highest predicted scores. In these graphs, nodes represent genes, and edges indicate regulatory direction, with arrows pointing from transcription factors to target genes. The predicted networks in most datasets show a “core-regulator–dominated” structure. For example, in the human embryonic stem cell dataset (hESC1000), TFAP2A emerges as a central hub connected to many downstream targets, suggesting that the model can identify key transcription factor–centered regulatory modules involved in development and differentiation.

**Fig 5:**
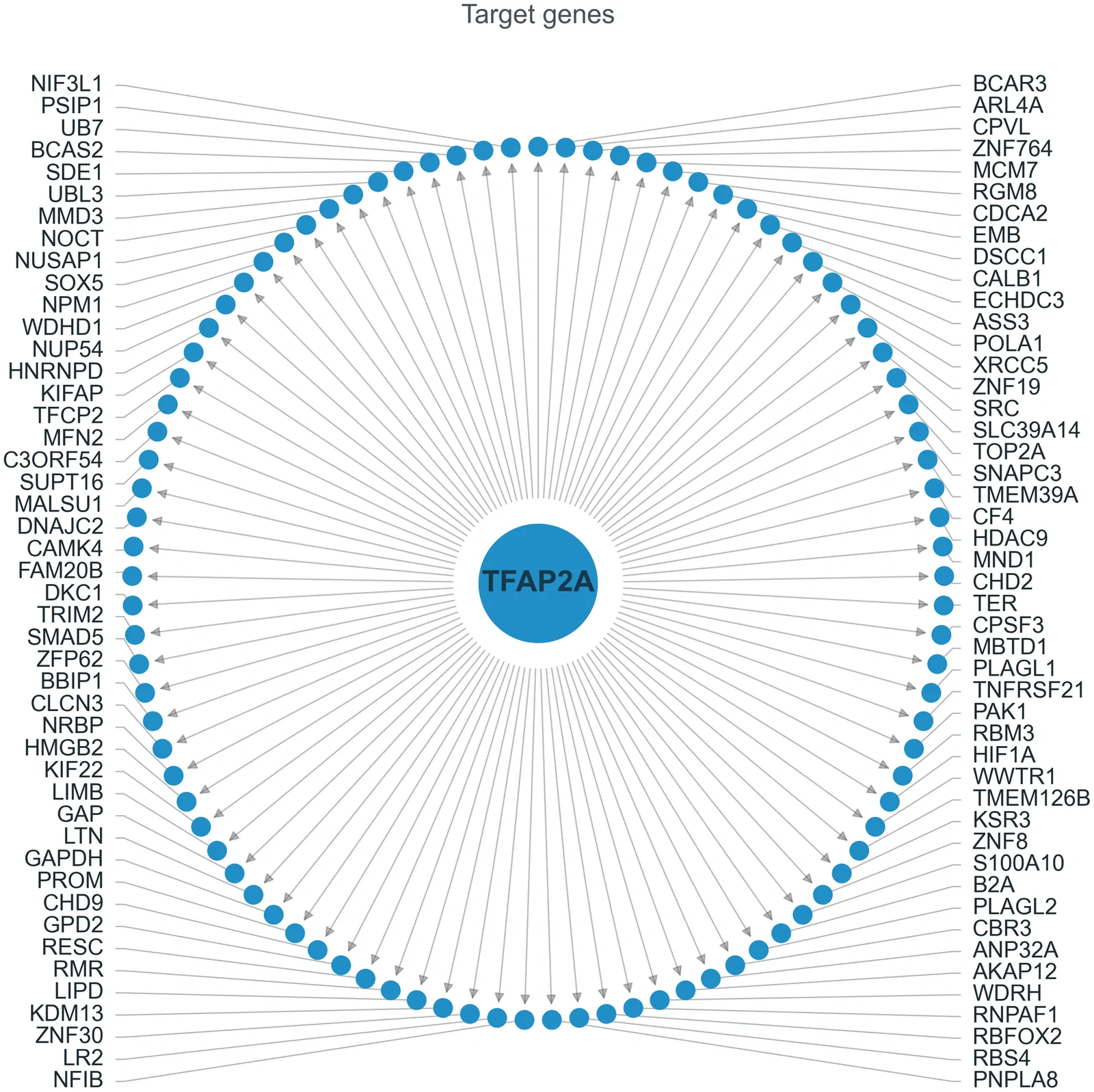
Directed visualization of the top-ranked TF–target regulations inferred from single-cell transcriptomic data. For each dataset, TF–target pairs were ranked by the model-predicted regulatory score, and the top 100 directed interactions were displayed for clarity. Arrows indicate the predicted regulatory direction (transcription factor → target gene), and node labels denote gene symbols (example shown: a TFAP2A-centered subnetwork).

Finally, the TF–target heatmap (Fig 6) presents predicted scores between transcription factors and target genes in a two-dimensional matrix. Yellow regions indicate strong regulatory relationships, while purple regions indicate weak or absent ones. Core factors such as TFAP2A, NANOG, and POU5F1 show consistently high predicted values across multiple targets, aligning with their known roles in pluripotency maintenance. These observations suggest that GP-DHT not only achieves superior quantitative performance but also captures biologically meaningful regulatory signatures.

**Fig 6:**
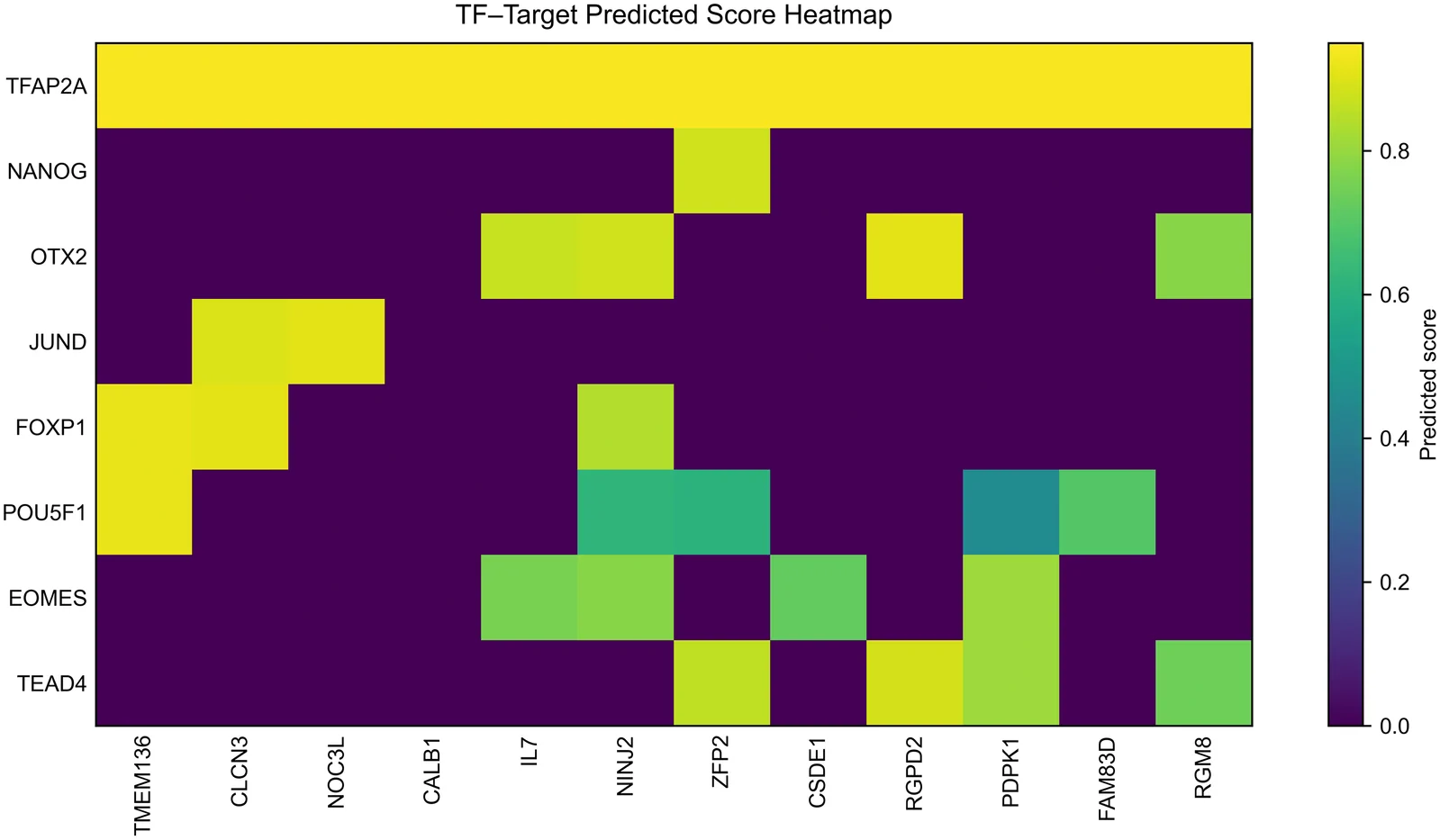
Heatmap of model-predicted regulatory scores for transcription factor–target gene pairs. Rows indicate transcription factors and columns indicate candidate target genes; each cell shows the predicted regulatory score (color scale), where higher values represent stronger predicted regulation / higher confidence. Low or near-zero scores indicate weak or unsupported regulatory associations among the displayed pairs.

In summary, GP-DHT demonstrates high accuracy and robustness for dataset-wise single-cell gene regulatory relationship prediction across the evaluated human and mouse datasets, while also showing strong interpretability in terms of network topology and biological relevance. This provides a reliable foundation for downstream functional validation and pathway analyses.

### 3.6 Auxiliary post-hoc attribution analysis

Beyond predictive performance, we further carried out an auxiliary post-hoc attribution analysis on the frozen GP-DHT model. For each candidate TF–target pair, the attribution module assigned directional importance weights to the two directed edges in the pair-level graph, and the mean of these edge weights was used as a pair-level attribution score. This score provided an explanatory ranking complementary to the original prediction probability.

As shown in Fig 7, the attribution-ranked regulatory network highlights TF-centered interactions that receive stronger explanatory support under the frozen predictor. Compared with ranking by prediction score alone, the attribution score helps distinguish highly confident pairs from those that are also more strongly supported in the post-hoc analysis.

**Fig 7:**
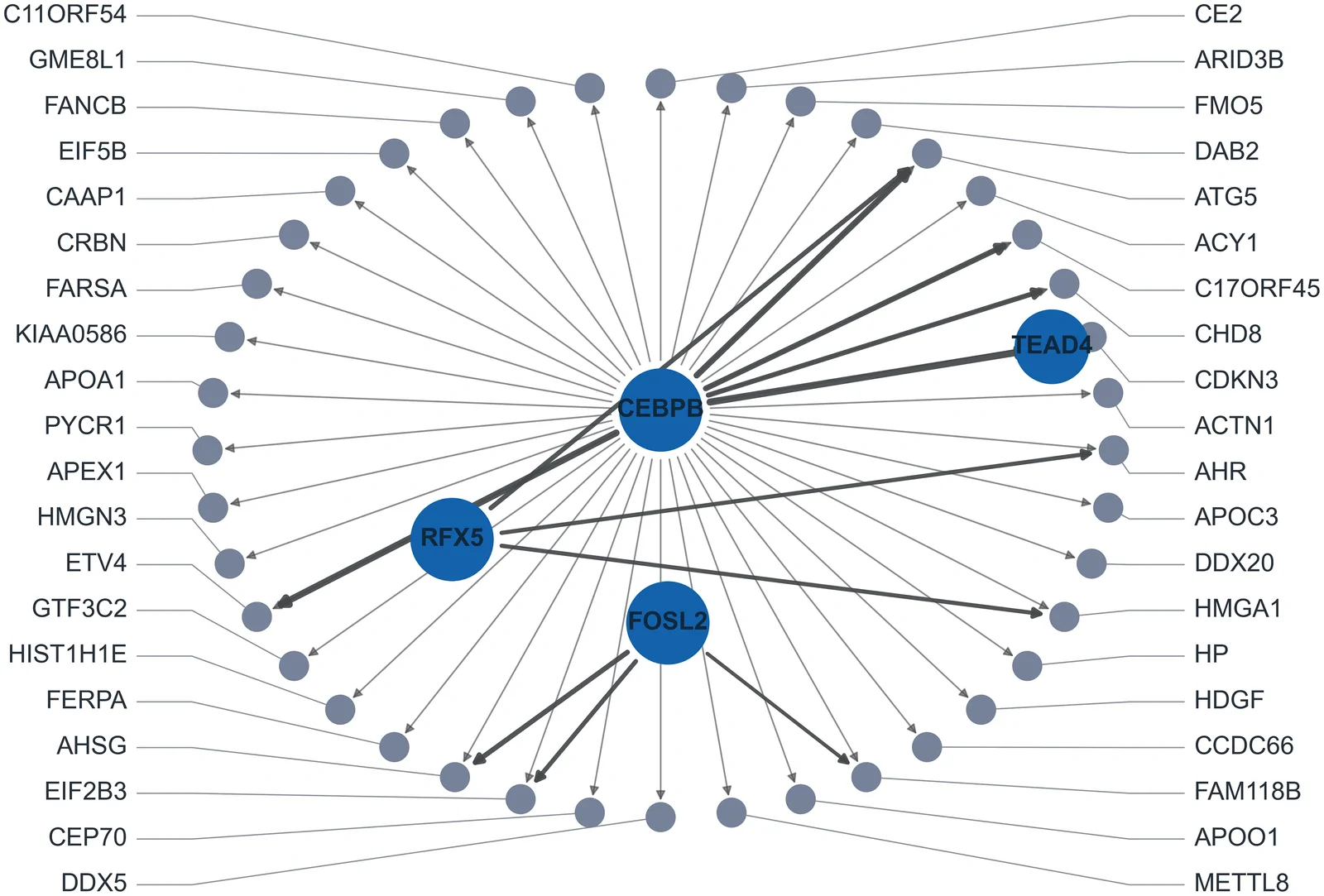
Top-K regulatory network ranked by pair-level attribution scores.

Fig 8 presents the attribution heatmap for representative TF–target pairs. High-value regions indicate directed edges that were consistently assigned larger importance weights across perturbed variants. This view provides a compact way to assess whether biologically relevant pairs remain prominent from both predictive and interpretive perspectives.

**Fig 8:**
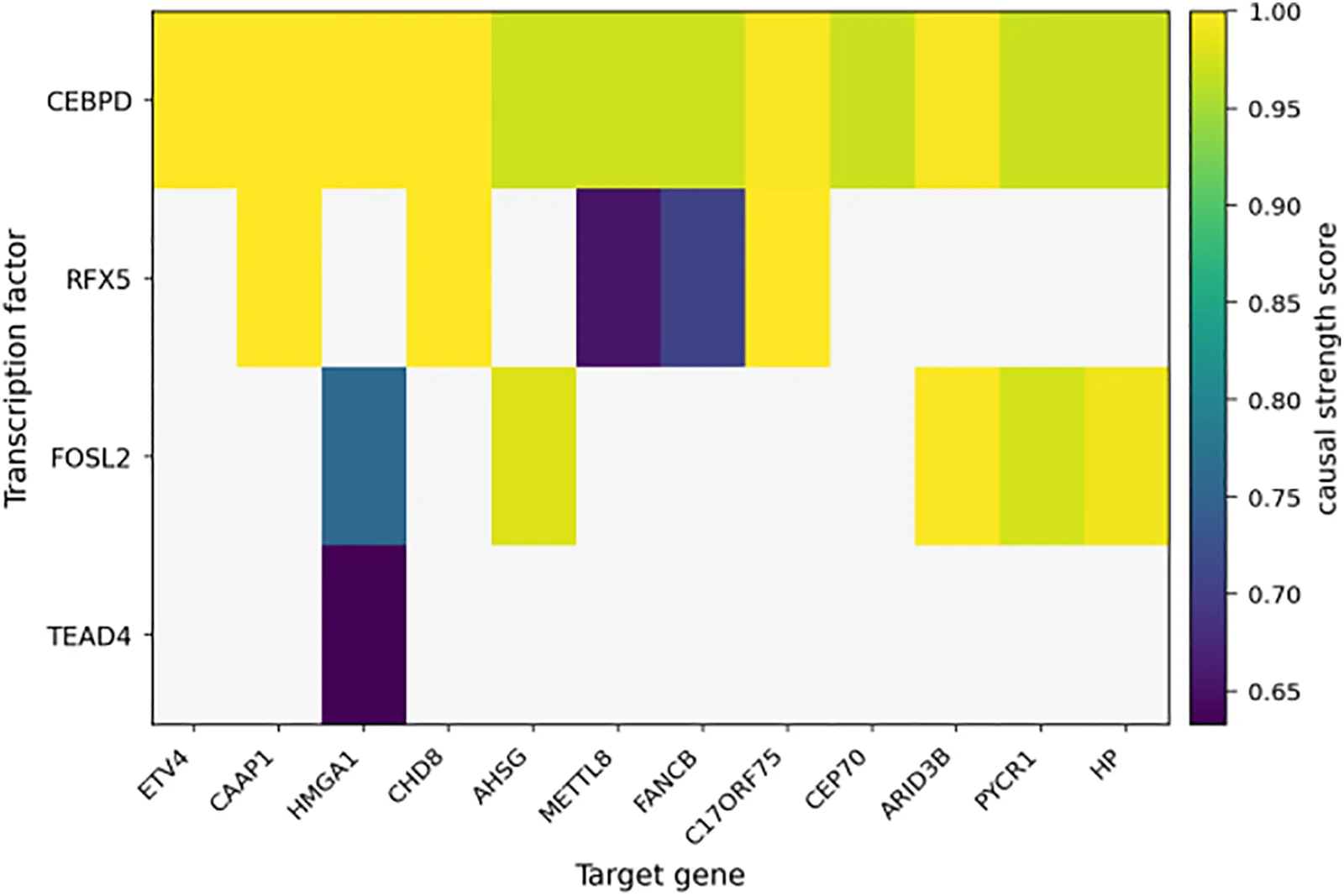
Heatmap of post-hoc pair-level attribution scores for representative TF–target pairs.

Table 4 summarizes representative TF-target pairs with high prediction scores and high attribution scores, together with available prior-support annotations. This table provides a concise qualitative bridge between model prediction and biological interpretation.

**Table 4:** Representative TF–target pairs with high prediction scores and high attribution scores.

rank	tf	target	attribution_score	regulatory_label
1	FOSL2	ID2	0.976	1
2	RFX5	EGF	0.975	1
3	EPAS1	TFAP2C	0.973	1
4	MYBL2	TFAP2C	0.969	1
5	TEAD4	TFAP2C	0.968	1
6	MAFF	CALML4	0.935	1
7	ARID2	HN1	0.919	1
8	ATF3	B3GNT7	0.917	1
9	BRCA1	PEMT	0.902	1
10	CEBPD	APEH	0.898	1
11	CEBPZ	KIAA0586	0.894	1
12	CREB1	DDX20	0.889	1

Overall, this auxiliary attribution analysis does not alter the main GP-DHT prediction pipeline, but provides an additional post-hoc layer for interpreting why specific gene-pair predictions are prioritized by the trained model.

### 3.7 Robustness and exploratory attribution diagnostics

To further contextualize model behavior beyond aggregate benchmark scores, we performed robustness stress tests and exploratory attribution diagnostics. These analyses were designed to characterize the sensitivity and stability of the trained predictor under controlled perturbations rather than to establish interventional causality.

As shown in Fig 9, the full GP-DHT model maintains the most favorable performance trajectory across sparsity, noise, and data-fraction stress conditions, supporting complementary contributions from the dual-head design and GenePairSupCon.

**Fig 9:**
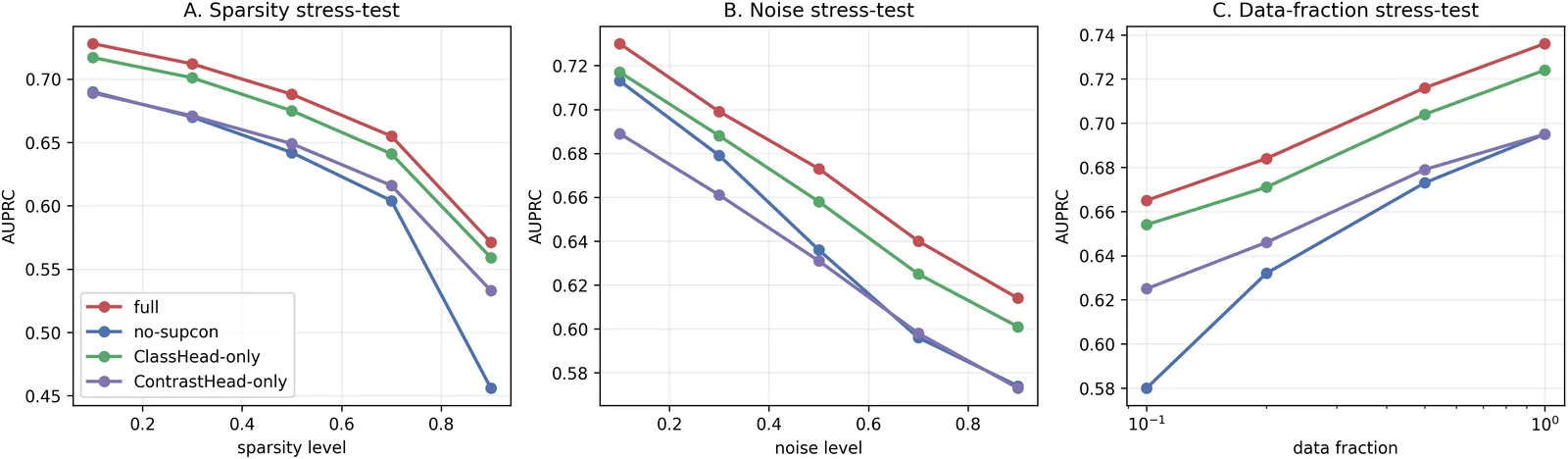
Component-wise robustness under sparsity, noise, and data-fraction stress tests. AUPRC trends are shown for the full GP-DHT model and ablated variants (without the supervised contrastive objective, classification-only head, and contrastive-only head). Across stress conditions, the full model maintains the strongest performance trajectory, supporting complementary contributions from the dual-head design and GenePairSupCon.

As further illustrated in Fig 10, supervised contrastive learning improves the geometry of the gene-pair embedding space by increasing the separation between positive and negative regulatory pairs. We then examined whether the attribution score was associated with the concentration of predictive signal.

**Fig 10:**
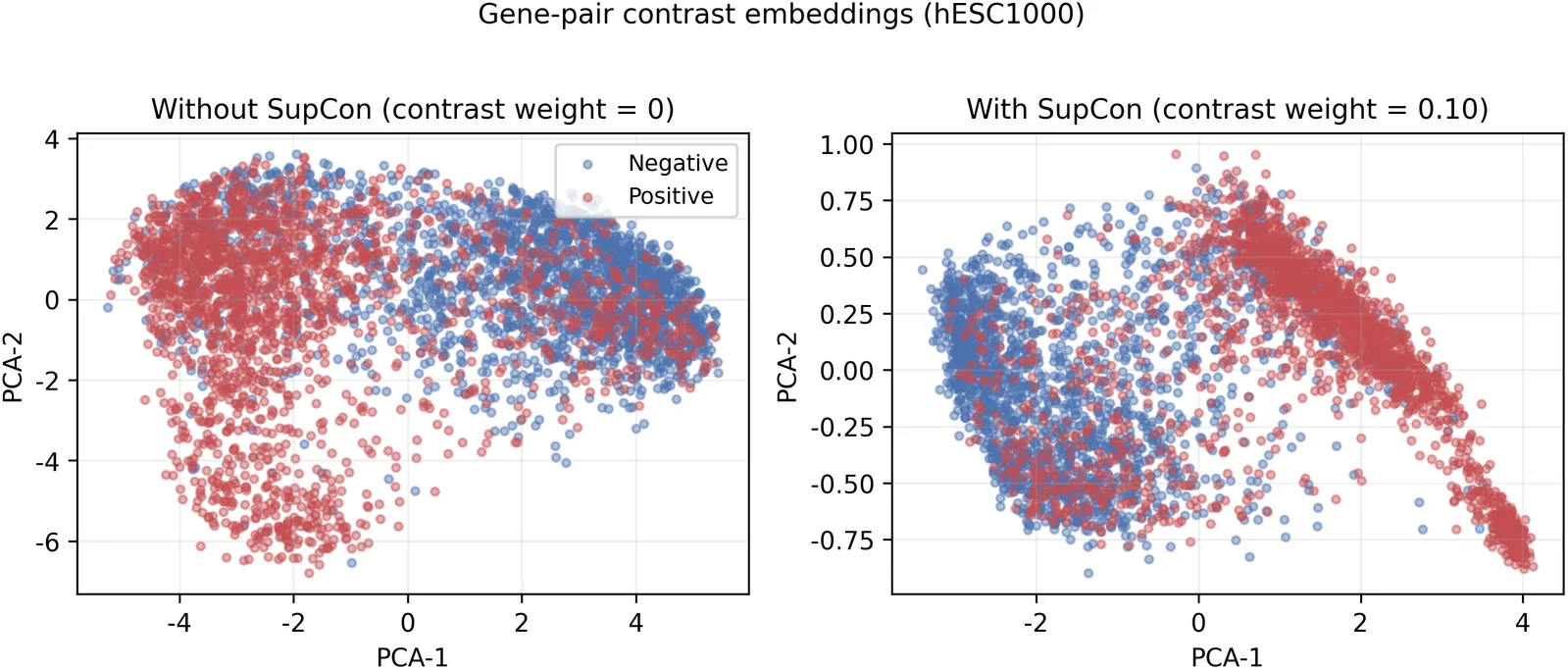
Gene-pair embedding geometry with and without supervised contrastive learning on hESC1000. Two-dimensional PCA projections of gene-pair representations show increased class-structure separation after introducing GenePairSupCon, consistent with improved discriminability between positive and negative regulatory pairs.

In the score-ranked sample-retention analysis (Fig 11), performance was evaluated on the retained subset after progressively keeping larger fractions of samples ranked by attribution score.

**Fig 11:**
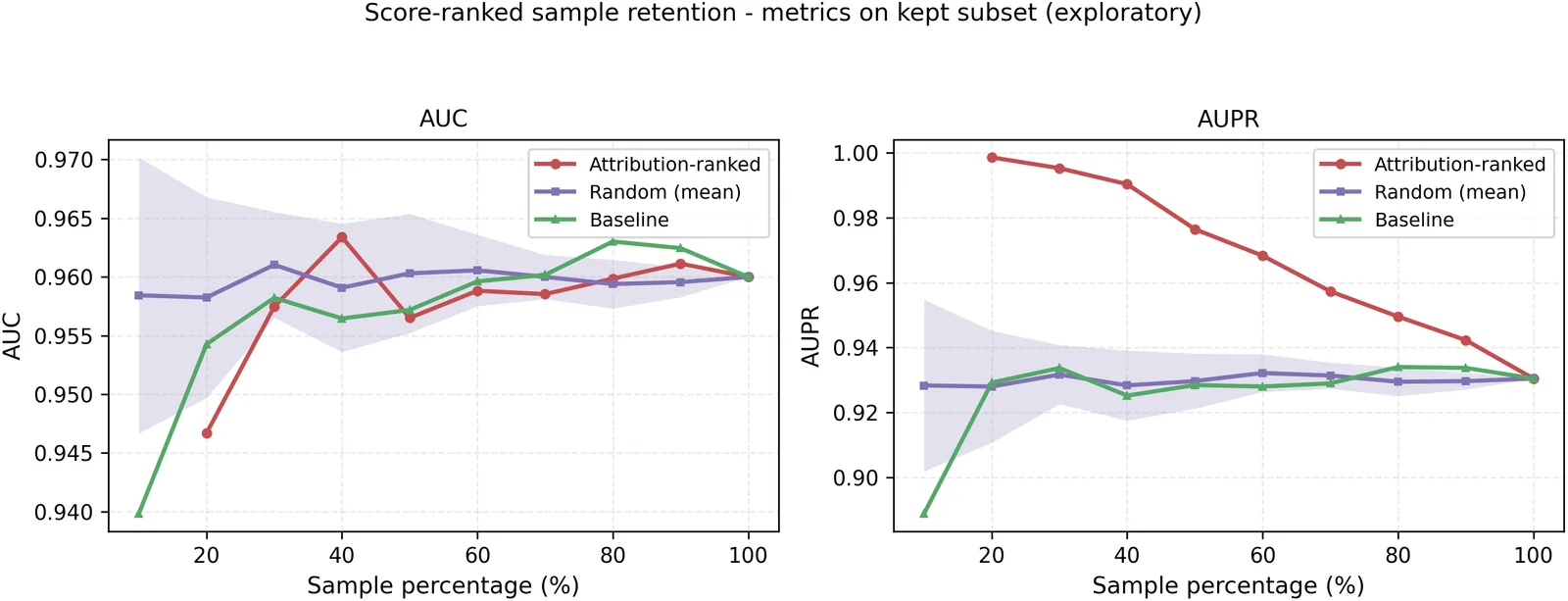
Score-ranked sample-retention analysis. Performance is evaluated on the retained subset after progressively keeping larger fractions of samples ranked by attribution score. This analysis probes score-associated concentration of predictive signal and should be interpreted as a sensitivity analysis rather than a formal attribution-fidelity evaluation.

In the complementary score-ranked sample-removal analysis (Fig 12), performance was evaluated on the remaining subset after progressively removing top-ranked samples.

**Fig 12:**
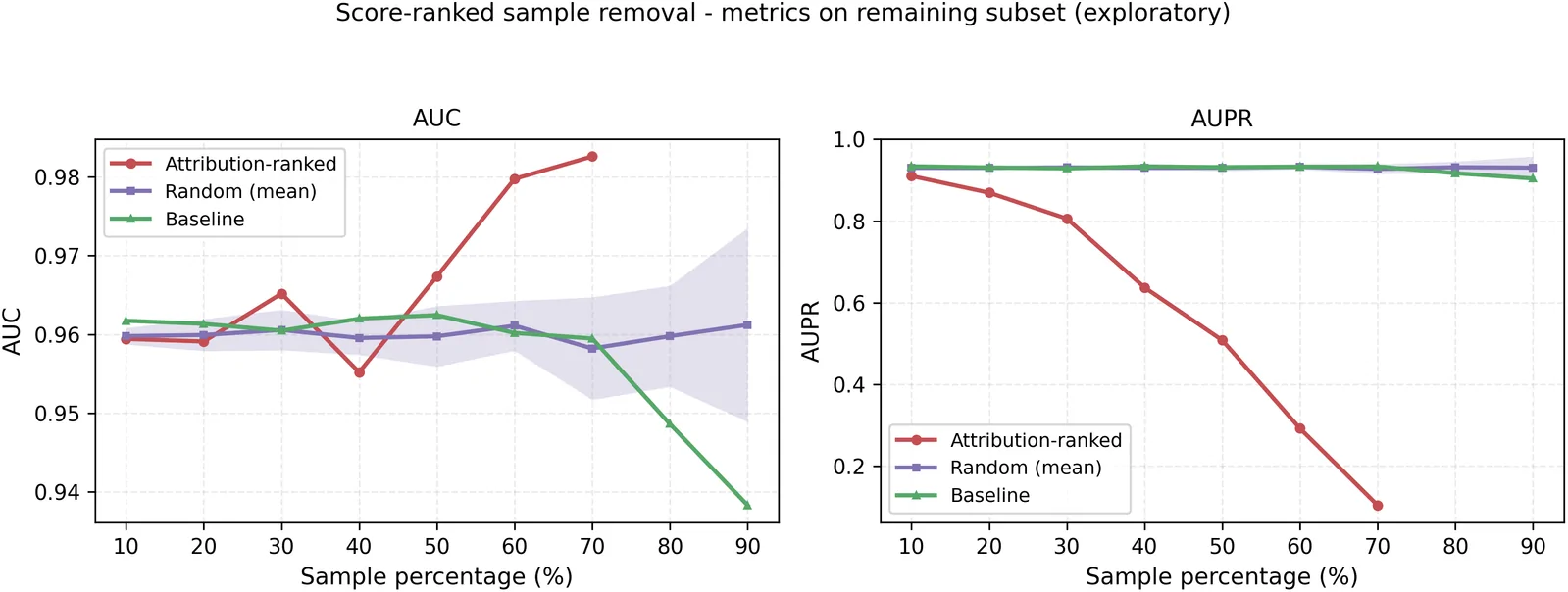
Score-ranked sample-removal analysis. Performance is evaluated on the remaining subset after progressively removing top-ranked samples. Differential degradation patterns across methods provide an additional view of score-concentration effects but do not by themselves constitute causal attribution evidence.

Finally, the reproducibility of the post-hoc attribution outputs across random seeds is summarized in Fig 13, which reports agreement, top-k overlap, and score dispersion statistics. Overall, these robustness and exploratory diagnostics are directionally consistent with the main benchmark findings: the full GP-DHT configuration remains more resilient under stress, and post-hoc explanatory signals exhibit measurable but finite stability. Accordingly, we treat the attribution outputs as decision-level support for model interpretation, complementary to but distinct from biological causal validation.

**Fig 13:**
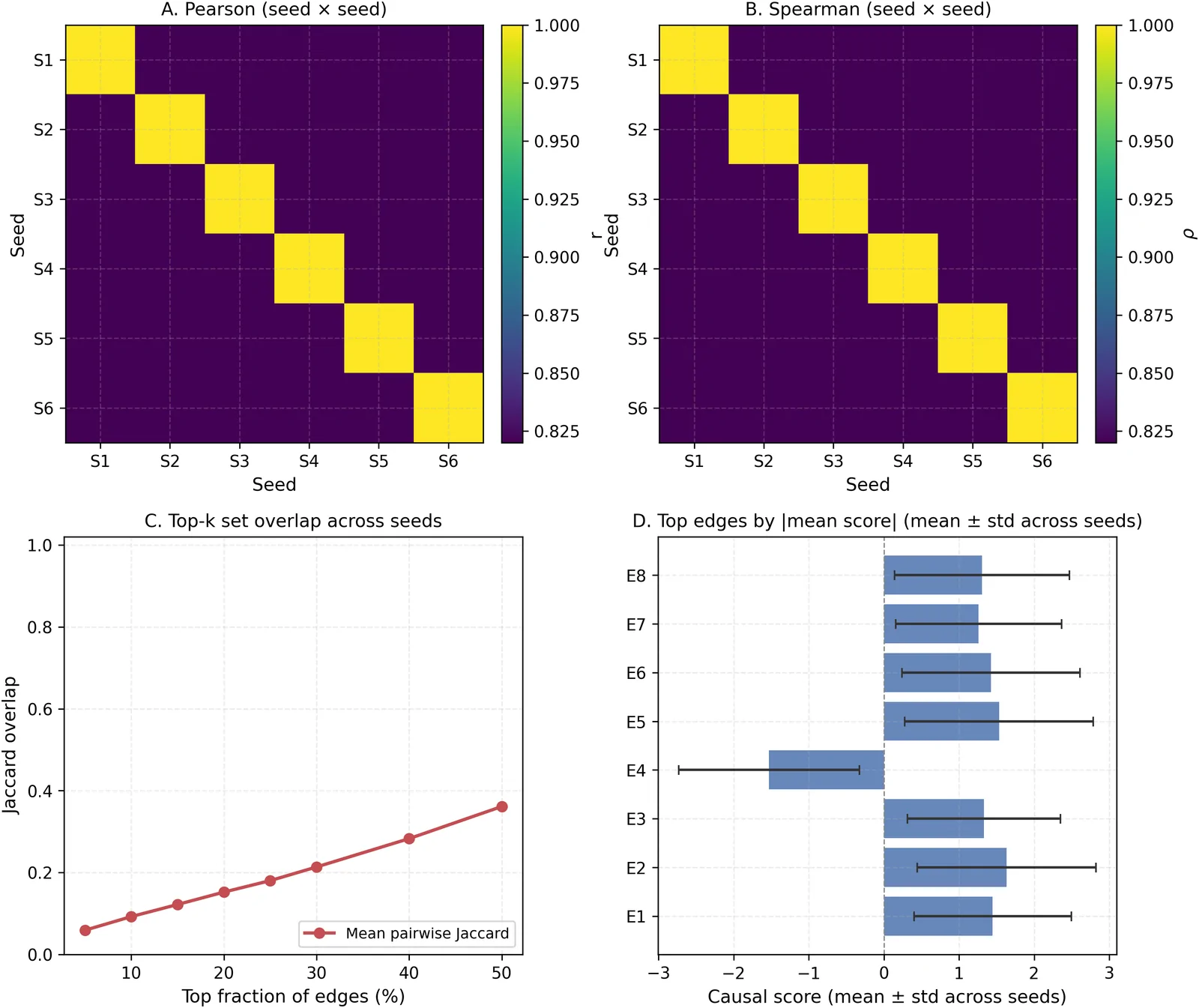
Seed-wise stability diagnostics of post-hoc attribution outputs. Pairwise Pearson and Spearman agreement, top-k overlap, and top-edge score dispersion are reported across random seeds. These plots provide a practical view of attribution reproducibility under a fixed training protocol and should be interpreted as stability diagnostics of the explanatory module.

## 4 Discussion

This study presents GP-DHT, a framework for dataset-wise single-cell gene regulatory network (GRN) inference with human–mouse shared-gene pretraining. The method is designed to address several practical challenges in single-cell GRN prediction, including expression noise, sparsity, incomplete regulatory labels, and the need for stable gene-pair representations. By integrating heterogeneous graph modeling, a dual-head Transformer module, and gene-pair-level supervised contrastive learning, GP-DHT provides an effective framework for modeling TF–target regulatory relationships from sparse single-cell transcriptomic data.

Extensive evaluations on seven benchmark datasets from the BEELINE framework demonstrate that GP-DHT consistently outperforms representative GRN inference methods, including HGATLink, GMFGRN, and GNN-Link, with improvements of approximately 5%–7% in both AUROC and AUPRC across most datasets. These performance gains suggest that explicitly modeling expression-gradient information and gene–cell heterogeneity helps extract regulatory signals under noisy and sparse conditions. In particular, the heterogeneous graph construction preserves multi-level expression relations that are often simplified or lost in conventional gene–gene graph formulations.

A central contribution of GP-DHT lies in its dual-head Transformer design, which separates regulatory prediction and representation regularization into two task-specific heads. The classification head directly optimizes TF–target regulatory link prediction, whereas the contrastive projection head applies GenePairSupCon to shape the gene-pair embedding space by increasing intra-class compactness and inter-class separation. Unlike GRN inference methods that rely mainly on a single prediction objective, GP-DHT couples discriminative link prediction with supervised contrastive representation learning. The quantitative embedding-quality analysis further supports this design: GenePairSupCon substantially improves Silhouette score, Davies-Bouldin index, Calinski-Harabasz score, and the inter/intra distance ratio, indicating that its main contribution is to improve gene-pair representation separability.

Ablation studies further validate the necessity of the proposed architectural components and design choices. In particular, the comparison between uniform and cumulative bucketing strategies highlights the importance of controlling edge redundancy in heterogeneous graphs. Uniform bucketing achieves a more favorable balance between representational efficiency and noise suppression by maintaining mutually exclusive expression levels, thereby improving downstream prediction accuracy. These findings suggest that careful discretization and relation design are important for effective graph-based modeling of single-cell transcriptomic data.

In addition to predictive performance, GP-DHT provides exploratory interpretive support through network visualization and post-hoc attribution analysis. Top-K regulatory subnetworks and TF–target heatmaps suggest that high-scoring predictions can form coherent TF-centered regulatory patterns, and the auxiliary attribution module provides decision-level support for prioritized TF–target pairs. These analyses may help identify candidate regulatory interactions or modules for downstream biological inspection. However, they should be interpreted as model-based exploratory evidence rather than direct proof of biological causality, because the present study does not include perturbation-based validation.

Despite its promising performance, GP-DHT has several limitations. First, the framework relies on static ChIP-seq-derived regulatory priors, which may not fully capture dynamic or context-specific regulatory changes. Incorporating time-resolved data, perturbation experiments, or causal intervention signals is therefore an important direction for future research. Second, the negative-label pairs used in the main HNS pipeline should be interpreted as putative negatives rather than experimentally verified non-regulatory pairs. The negative-label sensitivity analysis shows that stricter negative-label settings reduce model performance, indicating that negative-label construction materially affects task difficulty and that results based on easier negative candidates should be interpreted conservatively. Third, pooled human–mouse shared-gene pretraining should not be interpreted as direct species-to-species GRN transfer. In this study, shared-gene pretraining is used for representation initialization, whereas final supervised fine-tuning and evaluation are conducted within individual BEELINE datasets. Therefore, strict train-on-human/test-on-mouse or train-on-mouse/test-on-human generalization remains an open challenge. Finally, the computational cost associated with heterogeneous message passing and Transformer-based modeling may limit scalability to extremely large graphs. Future work may explore sparse attention mechanisms, subgraph sampling strategies, multimodal regulatory evidence, and perturbation-derived labels to improve both biological reliability and computational efficiency.

Overall, GP-DHT advances single-cell GRN inference by combining multi-relational gene–cell graph encoding, Transformer-based TF–target classification, and supervised contrastive regularization. The framework improves dataset-wise regulatory prediction under sparse and noisy single-cell settings, while the shared-gene pretraining component provides a practical representation-initialization strategy rather than a claim of direct cross-species regulatory transfer.

## 5 Conclusions

GP-DHT provides a robust and interpretable framework for dataset-wise single-cell GRN inference by unifying heterogeneous graph learning, Transformer-based modeling, and supervised contrastive optimization. By combining multi-relational gene-cell graph encoding, Transformer-based TF-target link classification, and gene-pair-level contrastive regularization, GP-DHT improves regulatory prediction under noisy and sparse single-cell settings while also offering a practical post-hoc explanatory layer for model interpretation. The human-mouse shared-gene pretraining component is retained as a representation-learning and initialization strategy, whereas strict species-to-species GRN transfer is not claimed as an established capability of the current study.

## Supporting information

S1 Table Summary of ortholog mapping and shared-gene-space construction used for pooled pretraining. (DOCX)

S2 Table Auxiliary negative-label sensitivity analysis under alternative negative construction settings. (DOCX)
