# Supplementary material for: GP-DHT: A dual-head transformer with supervised contrastive learning for shared-gene pretraining and single-cell GRN prediction
Source: PLoS One. 2026 Oct 6;21(10):e0359164. doi: 10.1371/journal.pone.0359164 (PMC13641368; doi:10.1371/journal.pone.0359164)
Supplement: Supplementary file 1 [file pone.0359164.s001.docx]

# Support

S1 Table. Summary of ortholog mapping and shared-gene-space construction used for pooled pretraining

| **Item** | **Value** |
| --- | --- |
| Mapping resource | Ensembl BioMart-derived MAP_NAME files |
| Total human genes (union) | 2,457 |
| Total mouse genes (union) | 4,223 |
| Shared genes (raw intersection) | 875 |
| Ambiguous one-to-many/many-to-one mappings | Excluded before final shared-gene construction |
| Clean one-to-one shared orthologs | 875 |
| Gene-symbol standardization | Performed before ortholog matching |
