# Supplementary material for: GP-DHT: A dual-head transformer with supervised contrastive learning for shared-gene pretraining and single-cell GRN prediction
Source: PLoS One. 2026 Oct 6;21(10):e0359164. doi: 10.1371/journal.pone.0359164 (PMC13641368; doi:10.1371/journal.pone.0359164)
Supplement: Supplementary file 2 [file pone.0359164.s002.docx]

# Support

S2 Table. Auxiliary negative-label sensitivity analysis under alternative negative construction settings.

| **Auxiliary co-expression threshold** | **Negative-label setting** | **AUROC (mean ± SD)** | **AUPRC (mean ± SD)** | **Precision@K (mean ± SD)** | **ΔAUROC vs. random** | **ΔAUPRC vs. random** | **Interpretation** |
| --- | --- | --- | --- | --- | --- | --- | --- |
| 0% | Random putative negatives | 0.8847 ± 0.0153 | 0.8073 ± 0.0166 | 0.742 ± 0.031 | Reference | Reference | Easier negative setting |
| 0% | Hard negatives | 0.8312 ± 0.0223 | 0.7538 ± 0.0273 | 0.681 ± 0.040 | -0.0535 | -0.0535 | More stringent setting |
| 5% | Random putative negatives | 0.8827 ± 0.0143 | 0.8053 ± 0.0170 | 0.735 ± 0.034 | Reference | Reference | Easier negative setting |
| 5% | Hard negatives | 0.8315 ± 0.0204 | 0.7541 ± 0.0248 | 0.670 ± 0.038 | -0.0512 | -0.0512 | More stringent setting |
| 10% | Random putative negatives | 0.8865 ± 0.0131 | 0.8091 ± 0.0131 | 0.749 ± 0.028 | Reference | Reference | Easier negative setting |
| 10% | Hard negatives | 0.8325 ± 0.0225 | 0.7551 ± 0.0283 | 0.687 ± 0.036 | -0.0540 | -0.0540 | More stringent setting |
